# Building Trust in Generics and Biosimilar: Strengthening Regulatory Confidence

**DOI:** 10.21315/mjms-10-2025-s03

**Published:** 2025-12-31

**Authors:** Mohammad Sharif Abu Hassan, Farshila Emran, Sharifah Fauziyah Syed Mohthar, Roziana Ab Rahim, Amielia Ab Hamid

**Affiliations:** 1 Hanan Medicare Sdn. Bhd., Rawang, Selangor, Malaysia; 2 Hanan Regulatory Services Sdn. Bhd., Rawang, Selangor, Malaysia

**Keywords:** generics, biosimilar, regulatory compliance, bioequivalence, medicine accessibility, medical industry, medicines, Malaysia

## Abstract

This article discusses the crucial role of traditional small-molecule generic drugs and biosimilar medicines in enhancing healthcare accessibility, particularly in lower- and middle-income countries such as Malaysia. It highlights how Malaysia’s evolving regulatory science, supported by the Drug Control Authority (DCA) and the National Pharmaceutical Regulatory Agency (NPRA), fosters an environment where quality, safety, efficacy, and affordability converge, aligning with national healthcare goals. Hanan Regulatory Services Sdn. Bhd. (HRS), the regulatory arm of the Hanan Group of Companies, upholds rigorous standards for product registration, bio-equivalence testing, manufacturing audits, pharmacovigilance and training. Malaysia’s regulatory framework requires the actual measured difference in bioavailability between the generic and innovator products to be on average, extremely small—approximately 3.5% only. These measures, along with post-marketing surveillance and good manufacturing practice, address misconceptions about generics, emphasising that their lower price does not equate to inferior quality.

On 4 March 2025, HRS registered Rapilin 30, Malaysia’s first premixed insulin aspart biosimilar, reflecting the growing industry maturity. In the United States, generics and biosimilars generated an estimated USD 467 billion in savings in 2024, demonstrating the economic potential of such medicines. Malaysia’s continued adherence to international regulatory standards and collaboration between public and private sectors will be key to sustaining confidence, achieving national pharmaceutical self-reliance and medicine security.

## Introduction

Traditional small-molecule generic drugs (generics) and biosimilar medicines play an increasingly vital role in strengthening healthcare accessibility, particularly in low-and middle-income countries. In Malaysia, the continued evolution of regulatory science has fostered a supportive environment where quality, safety, efficacy, and affordability converge to serve national healthcare goals aligned with the aspirations of the Dasar Ubat Nasional (DUNas).

At the centre of this effort stands Hanan Regulatory Services Sdn. Bhd. (HRS)— the regulatory arm of the Hanan Group of Companies—whose mission is to uphold the highest standards of regulatory compliance, scientific transparency, and patient safety. With more than 25 years of combined experience, HRS provides full-spectrum services covering product registration, pharmacovigilance, auditing, and training, ensuring that every product marketed under the Hanan brand as well as those of its customers, meets the stringent regulatory benchmarks.

Malaysia’s pharmaceutical landscape has grown more sophisticated in recent years. What used to be an industry dominated by innovator and generic medicines, has now opened its doors to more biosimilars being introduced in the country. As observed by Thao et al. (1), Malaysia’s biosimilar approval rate is steadily increasing, with regulatory turnaround times shortening in alignment with the European Medicines Agency. Such progress reflects not only improved regulatory efficiency but also the maturity of local pharmaceutical players who understand and embrace the scientific rigour required for complex biological medicines (1).

## Regulatory Excellence as the Core of Trust

Public trust in healthcare depends on the integrity of the healthcare system itself, which begins with strong regulatory oversight. Malaysia’s Drug Control Authority (DCA), supported by the National Pharmaceutical Regulatory Agency (NPRA) as its secretariat, ensures that all medicines manufactured, imported, and distributed within the country comply with the Control of Drugs and Cosmetics Regulations 1984 (CDCR 1984). The official regulatory approval by DCA, based on rigorous evaluation of scientific data for generic and biosimilar medicines, serves as an objective, expert-validated guarantee of interchangeability and regulatory compliance, ensuring the quality, safety, and efficacy of all medicines.

The NPRA also imposes strict adherence to current Good Manufacturing Practice (GMP) regulations. Key measures include requiring manufacturing sites, whether local or overseas, to undergo frequent and rigorous inspections by its highly professional inspectorates to ensure the requirements of GMP adherence in all process control aspects of production, from raw material quality, air handling systems, and equipment calibration to the training of personnel, ensuring every batch meets the required quality attributes.

Within this framework, Hanan Regulatory Services acts as both gatekeeper and facilitator. Its regulatory consultants provide expert support on product classification, dossier compilation, submission strategy, and compliance with current GMP and Good Clinical Practice (GCP). The team’s scope also extends to Good Distribution Practice, Good Pharmacovigilance Practice (GVP), enabling clients and internal stakeholders to maintain a robust chain of compliance from product development through post-marketing.

## Ensuring Quality, Safety, and Efficacy in Generics

Generic medicines remain the cornerstone of healthcare affordability. However, public misconceptions often persist, sometimes even amongst medical professionals—that equate lower price with inferior quality. Addressing this perception requires continuous education, transparent data, and consistent regulatory enforcement.

In Malaysia, generic products containing scheduled poisons in oral solid dosage forms must demonstrate bioequivalence (BE) to their reference products before they can be approved by DCA. This is measured through pharmacokinetic studies comparing the maximum concentration (C_max_) and the area under the curve (AUC) of both formulations. The difference in bioavailability between the two drugs should ideally lie within the therapeutic bioequivalence interval. The international bioequivalence standard requires that the 90% confidence interval for the ratio of the generic’s to the reference’s average AUC and C_max_ values fall entirely within the limits of 80% to 125%. For this strict statistical requirement to be met, the actual measured difference in bioavailability between the generic and innovator products is, on average, extremely small—approximately 3.5%.

HRS actively oversees this process, ensuring all studies are conducted at NPRA-accredited BE studies centres that comply with GCP and Good Laboratory Practice.

Post-approval, the work continues through pharmacovigilance and post-marketing activities. These ongoing assessments detect adverse trends early and support continuous benefit—risk evaluation, which sustains confidence among prescribers and patients alike.

## A National Milestone: Malaysia’s First Biosimilar Insulin Analogue Premix

With over 1,200 biosimilar approvals worldwide, these quality assured medicines are well positioned to reshape the treatment landscape. Each one represents an opportunity to optimise national spending and improve the lives of patients living with Non-Communicable Diseases (2).

On 4 March 2025, Malaysia achieved a significant milestone when Hanan Group, through HRS, successfully registered a biosimilar Insulin Aspart Mix 30/70, under the brand name Rapilin 30—the first biosimilar insulin of its kind to be registered by a Malaysian company.

Biosimilars, unlike traditional small-molecule generics, require advanced analytical characterisation, manufacturing precision, and comparative clinical data to establish biosimilarity with the innovator product. Beyond ensuring therapeutic equivalence and quality, HRS, together with its global drug safety unit, managed the product’s risk-management plan and other post-marketing safety commitments—all vital components for long-term confidence in biosimilar use.

Economically, the impact of biosimilars is equally profound. As highlighted in the U.S. Generic and Biosimilar Medicines Savings Report 2025 (3), generics and biosimilars together have contributed to global healthcare savings exceeding USD 467 billion, enabling patients and governments to reallocate funds toward innovation, prevention, and access (3). Such figures validate why Malaysia must continue to nurture the development of local biosimilar players while maintaining stringent regulatory stewardship.[Fig f1-01mjms3206_ed]

## Regulatory Compliance as a Culture

Regulatory compliance in the pharmaceutical industry is not a departmental function—it is a shared organisational value that influences decision-making at every level. Every product dossier, inspection report, and safety update represents more than a procedural requirement; it reflects an ethical responsibility to uphold patient wellbeing.

Within Hanan Group, this commitment is reinforced through continuous professional development, internal audits, and cross-functional collaboration, ensuring that compliance principles remain visible throughout every stage of the product lifecycle. The result is a corporate culture that equates regulatory excellence with brand integrity—a standard that has made Hanan Group synonymous with credibility in the Malaysian pharmaceutical sector.

This philosophy mirrors the broader transformation within Malaysia’s healthcare landscape: a shift from viewing regulation as an administrative hurdle to recognising it as a foundation of public trust. Through scientific transparency and strict alignment with NPRA and international regulatory guidelines, HRS helps ensure that every generic and biosimilar medicine registered in Malaysia is not only of assured quality, safety, and efficacy, but is also cost-effective and contributes to long-term medicine security.

## Conclusion

Malaysia’s regulatory framework, particularly in the area of generic medicines and biosimilars, meets stringent international standards of quality, safety, and efficacy. The robust enforcement of regulations within the generic and biosimilar industry, combined with integrated regulatory oversight, continues to build trust among healthcare professionals and the public—ensuring that generic and biosimilar medicines remain a safe, effective, and affordable option for patients nationwide.

Malaysia’s aspiration to become a regional leader in generic and biosimilar medicines depends on its ability to balance innovation, affordability, and compliance. As the nation moves towards pharmaceutical self-reliance and medicine security, regulatory institutions and private-sector partners must continue collaborating to strengthen oversight, capacity-building, and transparency.

## Figures and Tables

**Figure 1 f1-01mjms3206_ed:**
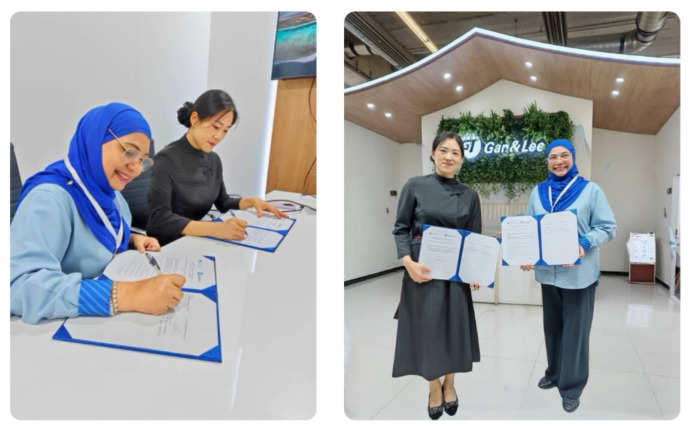
MOU signing between Hanan Medicare Sdn. Bhd and Gan & Lee on 28 October 2025 at Convention on Pharmaceutical Ingredients (CPHI) Frankfurt
